# Nephrolithiasis and risk of hypertension: a meta-analysis of observational studies

**DOI:** 10.1186/s12882-017-0762-8

**Published:** 2017-11-29

**Authors:** Weifeng Shang, Yuanyuan Li, Yali Ren, Yi Yang, Hua Li, Junwu Dong

**Affiliations:** 1 Department of Nephrology and Rheumatology, The Forth Hospital of Wuhan Affiliated with Tongji Medical College, Huazhong University of Science and Technology, Wuhan, Hubei 430030 People’s Republic of China; 2 Department of Respiratory Medicine, The Forth Hospital of Wuhan Affiliated with Tongji Medical College, Huazhong University of Science and Technology, Wuhan, Hubei 430030 People’s Republic of China; 3 Department of Medical Affaires, Liyuan Hospital Affiliated to Tongji Medical College, Huazhong University of Science and Technology, Wuhan, Hubei 430030 People’s Republic of China; 4 Department of Nephrology, Tongji Hospital Affiliated to Tongji Medical College, Huazhong University of Science and Technology, Wuhan, Hubei China

**Keywords:** Kidney stone, Nephrolithiasis, Hypertension, Meta-analysis

## Abstract

**Background:**

Observational studies have demonstrated an association between nephrolithiasis and hypertension. The aim of this meta-analysis was to summarize all available evidence.

**Methods:**

PubMed, EMBASE, the Cochrane Central Register of Controlled Trials databases, and the reference lists of relevant articles were searched to identify observational studies that reported study-specific risk estimates comparing the risk of hypertension in patients with nephrolithiasis. We used a random-effect model to pool the study-specific risk estimates. We also assessed the potential heterogeneity by subgroup analyses, meta-regression analyses, and sensitivity analyses.

**Results:**

A total of 7 articles including 9 studies (*n* = 313,222 participants) were eventually identified in this meta-analysis. In comparison with the patients who did not have nephrolithiasis, nephrolithiasis significantly increased the risk of hypertension (OR, 1.43; 95% CI, 1.30–1.56), with significant heterogeneity between these studies (*I* ^2^ = 83.5%, *P* <0.001). The heterogeneity reduced in subgroups of cohort studies, USA, large sample size trials, men, and adjustment for confounding factors ≥ 5. Sensitivity analysis further demonstrated the results to be robust.

**Conclusions:**

Nephrolithiasis is associated with increased risk of hypertension. Future randomized, high-quality clinical trials are encouraged to definitively clarify the relationship between nephrolithiasis and hypertension, which may influence clinical management and primary prevention of hypertension in nephrolithiasis patients.

**Electronic supplementary material:**

The online version of this article (10.1186/s12882-017-0762-8) contains supplementary material, which is available to authorized users.

## Background

Nephrolithiasis is a common condition, with the prevalence varying by age and sex. The disease usually presents in men aged 60 to 69, with a prevalence rate of approximately 1.7 to 8.8% worldwide [1, 2]. Hypertension is defined as persistent elevation of systematic arterial blood pressure (systolic pressure ≥ 140 mmHg and/or diastolic pressure ≥ 90 mmHg). It is an extremely common cardiovascular disease, affecting over 30% of young adults and 70% of elderly individuals [3]. Hypertension is also a silent yet dangerous disease. Despite intensive studies aimed at identifying risk factors for hypertension, the exact pathogenic mechanisms of hypertension often are unclear. Nevertheless, there is growing evidence supporting that nephrolithiasis largely contribute to the occurrence of hypertension. Since the association between arterial hypertension and nephrolithiasis was described in 1965 for the first time by Tibblin [4], much effort has been devoted to this field. Data from several observational studies suggested a risk of hypertension in nephrolithiasis patients of 1.24–1.96 compared to the general population [5–11]. A previous review performed by Cupisti et al. [12] has shown the current understanding of the potential link between nephrolithiasis and the occurrence of hypertension, but no meta-analysis has been used to examine the relationship.

Given the fact that individual studies may have insufficient statistical power because of sample size, we performed a meta-analysis to collect all beneficial evidence to assess the risk of hypertension among nephrolithiasis patients, which may emphasize the importance of considering additional intervention methods in this area.

## Methods

Our study was conducted in accordance with the Preferred Reporting Items for Systematic Reviews and Meta-Analyses (PRISMA) statement checklist [13].

### Search strategy

PubMed, EMBASE, and Cochrane Library databases were searched for observational studies to March 18, 2017. The used search terms were as follows: “renal stones” or “renal stone” or “kidney stones” or “kidney stone” or “nephrolithiasis” or “calculi” and “hypertension” or “blood pressure” and “risk” or “incidence” or “epidemiology”. Furthermore, we searched reference lists of all included studies for additional eligible studies. Two of the authors (WS and YL) independently screened titles and abstracts, analyzed full-text articles, and ascertained the final eligible records. Conflicting results were resolved by discussion. We merged retrieved citations using EndNote X7.

### Inclusion and exclusion criteria

The inclusion criteria were: (1) the study design was a cross-sectional, case–control, or cohort study; (2) identified nephrolithiasis as exposure, including medical records, questionnaire, direct interview etc.; (3) the outcome measure was hypertension, including medical records, questionnaire, blood pressure measurement, direct interview etc.; and (4) odds ratio (OR) or hazard ratio (HR) or risk ratio (RR), and the corresponding 95% confidence interval (CI) were reported or could be calculated. Reviews, letters, case reports, and animal studies were excluded.

### Data extraction

Study characteristics were extracted by two authors (WS and YL) separately as follows: first author’s name, publication year, country origin, study design, sample, average age, proportion of men, method of nephrolithiasis and hypertension diagnosis, and adjustment factors. When needed, we contacted the original author for clarification.

### Quality assessment

We evaluated the quality of studies using the Strengthening the Reporting of Observational Studies in Epidemiology (STROBE) statement [14]. Two authors (WS and YR) performed the quality assessment independently and disagreements were resolved by discussion.

### Statistical analyses

The study-specific most adjusted HR, RR or OR was used to compute a summary OR and its 95%CI. HRs and RRs were directly considered as ORs [15]. Heterogeneity of ORs among studies was assessed using the Chi-squared based on Q-statistic test (*P* < 0.10) and quantified by I^2^ statistic. I^2^ values were considered to represent insignificant (0–25%), low (26–50%), moderate (51–75%), and high (>75%) heterogeneity [16]. The random-effects model was used to calculate the combined risk estimates. Subgroup analysis and univariable random effects meta-regression were further conducted to explore the potential source of heterogeneity. Stratified analyses were conducted based on study design (cohort or cross-sectional), region (USA or non-USA), sample size (<35,000 or ≥35,000), gender (men or women), and the number of confounders adjusted for (<5 or ≥5). We conducted sensitivity analyses to assess the influence of a study on the pooled effect estimate by recalculating the pooled OR with removal of one study in each turn. Reporting bias was evaluated using Egger’s test [17]. All meta-analyses were performed by the STATA (version 10.0, Stata Corporation, College Station, TX, USA). *P* < 0.05 in 2-tailed test was considered to be statistically significant.

## Results

### Study selection, characteristics, and quality

**Fig. 1:**
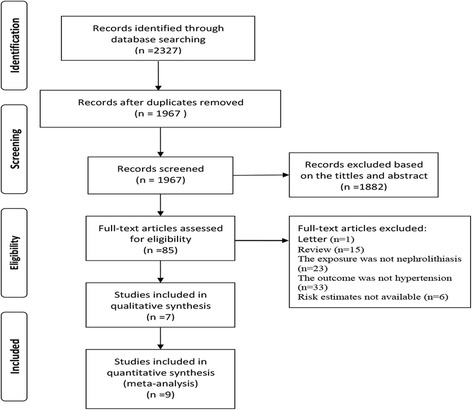
Flow chart of study selection

As shown in Fig. 1, our literature search returned 2327 results for relevant articles, and the full text retrieved for 85 articles. Finally, we identified 9 observational studies, based on 7 articles.

The main characteristic of the studies included are presented in Table 1. Included studies were published during 1998–2017. These articles included 4 cohort studies, and 5 cross-sectional studies. Of these studies, six were conducted in United States, one in Italy, one in Portugal, and one in Japan. The primary analysis included data for 313,222 participants derived from 9 observational studies that reported an association between nephrolithiasis and the risk of hypertension.

**Table 1:** Characteristics of the identified studies

First author Year	Country	Design	Sample	Average age (y)	Men (%)	Definition of Kidney stones	Confirmation of Hypertension	Adjustments
Madore et al. (1) 1998(cohort)	USA	cohort	67,745	51.5	0.0%	medical records	physician diagnosed hypertension	age (in 5-year categories),BMI (in quintiles), dietary intake of calcium, sodium, potassium, magnesium, and caffeine (in quintiles), and intake of alcohol (in eight categories)
Madore et al. (1) 1998(cross-sectional)	USA	cross-sectional	89,376	NA	0.0%	medical records	physician diagnosed hypertension	age
Madore et al. (2) 1998(cohort)	USA	cohort	37,809	53.2	100.0%	initial questionnaire	physician diagnosed hypertension	age, BMI and the intake of calcium, sodium, potassium, magnesium, and alcohol
Madore et al. (2) 1998(cross-sectional)	USA	cross-sectional	51,529	NA	100.0%	initial questionnaire	physician diagnosed hypertension	age
Strazzullo et al. 2001 [7]	Italy	cohort	381	45.1	100.0%	fixed-sequence questionnaire	examination	age
Gillen et al. 2005 [8]	USA	cross-sectional	20,029	43.6	46.6%	patient reported	self-reported previous diagnosis of hypertension, SBP, DBP, and pulse pressure calculated as the difference between SBP and DBP	age, sex, race (African American versus other), BMI, history of CVD (myocardial infarction, stroke, congestive heart failure), diabetes, and smoking status (ever versus never), dietary intake, insurance status, alcohol use, household income, and marital status.
Domingos et al. 2011 [9]	Portugal	cross-sectional	23,349	NA	NA	direct interview	direct interview	age and BMI
Ando et al. 2012 [10]	Japan	cross-sectional	20,990	NA	NA	NA	NA	overweight, hypertension and hyperuricemia
Kittanamongkolchai et al. 2017 [11]	USA	cohort	2014	41.5	52.9%	chart review	medical records	age, sex, BMI, serum creatinine, CKD, diabetes, gout, coronary artery disease, dyslipidemia, tobacco use, and alcohol abuse

Note: *NA* not avaliable, *BMI* body mass index, *CVD* cardiovascular disease, *CKD* chronic kidney disease, *SBP* systolic BP, *DBP* diastolic BP

According to the STROBE, all but one included studies were of high quality (Additional file 1: Table S1).

### Nephrolithiasis and risk of hypertension

**Fig. 2:**
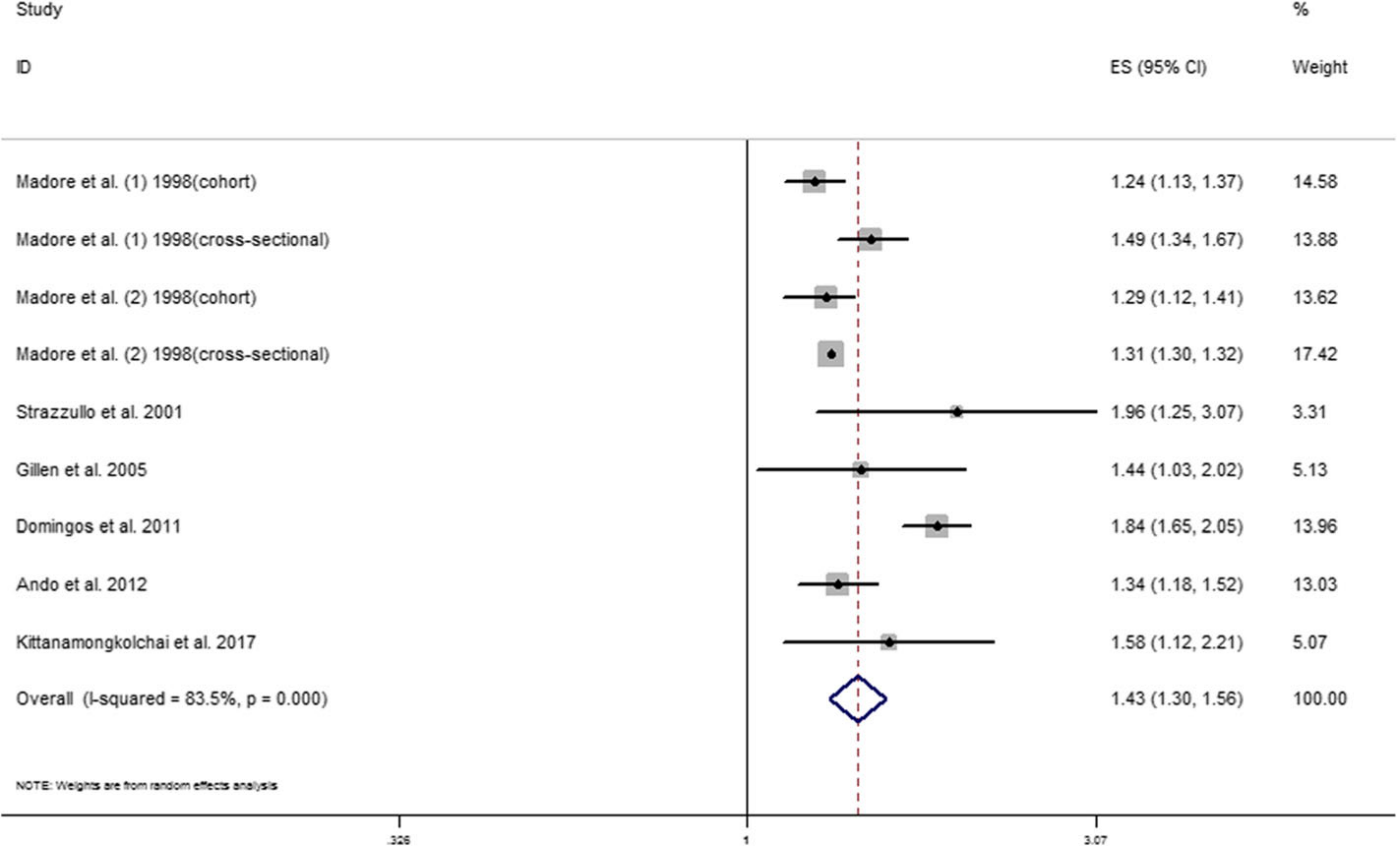
Risk of hypertension in nephrolithiasis compared with controls

As shown in Fig. 2, the multivariate-adjusted OR of hypertension within the 9 individual study populations ranged between 1.24 and 1.96, with an overall multivariate-adjusted OR of 1.43 (95% CI, 1.30–1.56). Significant heterogeneity was observed (*I* ^2^ = 83.5%, *P* <0.001).

### Subgroup analyses

In most cases, low-to-high heterogeneity was still present in stratified analyses unless adjustment for confounding factors was more than 5 (*I* ^2^ = 0%). We used meta-regression to explore the sources of heterogeneity and found that study design, resign, sample size, gender, and adjustment for confounding factors may be potential sources of heterogeneity (Table 2).

**Table 2:** Subgroup analyses of hypertension in patients with kidney stones

Subgroup	No. of studies	OR (95% CI)	I^2^ (%)	*P* ^a^	*P* ^b^
Study design					
Cohort	4	1.33 (1.18, 1.49)	43.8	0.148	0.518
Cross-sectional	5	1.47 (1.28, 1.70)	90.7	<0.001	
Region					
USA	5	1.33 (1.26, 1.40)	37.8	0.154	0.106
Non-USA	3	1.64 (1.26, 2.14)	86.3	0.001	
Sample size					
< 35,000	5	1.59 (1.32, 1.92)	73.4	0.005	0.079
≥ 35,000	4	1.32 (1.25, 1.40)	54.3	0.087	
Gender					
Men	4	1.31 (1.25, 1.37)	13.4	0.326	0.547
Women	3	1.43 (1.21, 1.69)	78.2	0.010	
Adjustment for confounding factors					
< 5	5	1.51 (1.30, 1.76)	91.2	<0.001	0.275
≥ 5	4	1.32 (1.14, 1.52)	0	0.497	

*OR* odds ratio, *CI* confidence interval

^a^ *P* value for heterogeneity among studies assessed with Cochran’s Q test

^b^ *P* value for interaction evaluated by meta-regression models

### Sensitivity analyses and reporting bias

Sensitivity analyses were performed by excluding one study at a time. And they indicated that the omission of any of the studies led to changes in estimates between 1.34 (95% CI: 1.27–1.40) and 1.47 (95% CI: 1.32–1.64). The changes were not significant. However, deletion of the Domingos et al.’s study reduced the heterogeneity from high to low levels (Additional file 2: Table S2). The *P* value of Egger’s test was 0.134, suggesting that there was no publication bias statistically.

## Discussion

To the best of our knowledge, this study is the first meta-analysis to present hypertension risk in patients with a history of nephrolithiasis. We confirmed nephrolithiasis was associated with an increased risk of hypertension. The risk of hypertension remained pronounced in all subgroups.

As with other published meta-analyses of this type [18, 19], our study has a high level of heterogeneity. We constructed subgroup analyses and meta-regression analyses to explore sources of heterogeneity. In subgroup analyses, the heterogeneity reduced in patients within cohort studies, USA, large sample size trials, men, and adjustment for confounding factors ≥ 5. This conclusion is supported by the results of the meta-regression, which showed that study design, resign, sample size, gender, and adjustment for confounding factors may be potential sources of heterogeneity. In addition, different follow-up time and adjust factors may be also the source of heterogeneity.

Note that female patients with kidney stones showed much higher risk for hypertension than male patients in our study. The underlying pathophysiology remains unclear. However, the differences by sex are not infrequent. A similar finding has also been observed in a meta-analysis on the association of nephrolithiasis and risk of incident chronic kidney disease (CKD) [18]. Similarly, a review also demonstrated a statistically significant increased risk of coronary heart disease in female patients with prior nephrolithiasis, but there was no significant association in male patients with prior nephrolithiasis [20]. Of note, Madore et al.’s study indicated that both women and men with hypertension at baseline were not more likely to develop nephrolithiasis during the follow-up [5, 6]. Due to the limited data, further studies are needed to direct the sex difference in hypertension response to nephrolithiasis.

The relation between nephrolithiasis and hypertension is rather unclear, but after our complete literature retrieval, we found several potential reasons which may explain the observed associations. First, alterations in calcium metabolism maybe have an important role in the pathogenesis of both nephrolithiasis and hypertension [21, 22]. Second, the traits of metabolic syndrome are factors highly prevalent in hypertensives as well as in kidney stone formers, so insulin resistance may be a common pathophysiological mechanism [23, 24]. Third, CKD is a condition which may occur more frequently in nephrolithiasis patients and in hypertensive patients. Therefore, CKD may be another factor involved in the linkage between nephrolithiasis and hypertension. Finally, inflammation and oxidative stress have been recently hypothesized as possible links between stone disease and hypertension [25]. Obviously, all of these potential reasons are comorbidities in nephrolithiasis and hypertension. However, more medical research is needed to explore and test the relevant presumption.

Several limitations of this meta-analysis should be pointed out. First, significant heterogeneity was detected in the nephrolithiasis and hypertension, the differences in characteristics of populations, study designs, sample size, men (%), diagnostic criteria, and adjusted confounders may contribute to the high heterogeneity. For example, the diagnosis of hypertension was inferred from self-reported blood pressure or patient questionnaire in two cross-sectional studies (Gillen et al. [8] and Domingos et al. [9]), which may bias the true incidence of hypertension. Second, we had no access to the information on the total number or type of nephrolithiasis. Therefore, we could not evaluate the association between different types of nephrolithiasis and hypertension. Third, most of the studies included were partially representatives of western countries, and thus extrapolating results to other parts of the world should be interpreted cautiously. Fourth, no publication bias was detected statistically in our study, but potential publication bias could not be completely ignored, given the fact that studies with null results tend not to be published. Finally, although all the included studies controlled for several known risk factors for hypertension, residual confounding cannot be excluded because the results of our study were based on observational studies.

## Conclusions

Our study demonstrates that nephrolithiasis is significantly associated with increased risk of hypertension. Well-designed randomized controlled trials are necessary to elucidate the underlying mechanism and will provide more effective preventive and therapeutic measures. Our study has important implications for public health, which emphasizes that clinicians pay attention to the potential association between nephrolithiasis and hypertension.

## Additional files

Additional file 1: Table S1. Strengthening the Reporting of Observational Studies in Epidemiology (STROBE) statement scores of the included published studies. (DOCX 17 kb)

Additional file 2: Table S2. Sensitivity analysis. (DOC 28 kb)

## Abbreviations

CI: 95% confidence
HR: Hazard ratio
NOS: Newcastle-ottawa scale
OR: Odds ratio
PRISMA: Preferred reporting items for systematic reviews and meta-analyses
RR: Risk ratio
STROBE: Strengthening the Reporting of Observational Studies in Epidemiology
