# Supplementary material for: Nephrolithiasis and risk of hypertension: a meta-analysis of observational studies
Source: BMC Nephrol. 2017 Nov 29;18:344. doi: 10.1186/s12882-017-0762-8 (PMC5708110; doi:10.1186/s12882-017-0762-8)
Supplement: Supplementary file 1 — Strengthening the Reporting of Observational Studies in Epidemiology (STROBE) statement scores of the included published studies. (DOCX 17 kb) [file 12882_2017_762_MOESM1_ESM.docx]

| Table S1 Strengthening the Reporting of Observational Studies in Epidemiology (STROBE) statement scores of the included published studies. | | | | | | | | | | | | | | | | | | | | | | | | | | | | | | | | | | | |
| --- | --- | --- | --- | --- | --- | --- | --- | --- | --- | --- | --- | --- | --- | --- | --- | --- | --- | --- | --- | --- | --- | --- | --- | --- | --- | --- | --- | --- | --- | --- | --- | --- | --- | --- | --- |
| Study | Title and abstract | | Introduction | | Methods | | | | | | | | | | | | | | Results | | | | | | | | | | | Discussion | | | | Other information | Total score |
|  | 1 | | 2 | 3 | 4 | 5 | 6 | | 7 | 8 | 9 | 10 | 11 | 12 | | | | | 13 | | | 14 | | | 15 | 16 | | | 17 | 18 | 19 | 20 | 21 | 22 |  |
|  | a | b |  |  |  |  | a | b |  |  |  |  |  | a | b | c | d | e | a | b | c | a | b | c |  | a | b | c |  |  |  |  |  |  |  |
| Madore et al. 1998(women) | 1 | 1 | 1 | 1 | 1 | 1 | 1 | NA | 1 | 1 | 1 | 1 | 1 | 1 | 1 | NA | NA | 0 | 1 | 1 | 0 | 1 | 0 | 1 | 1 | 1 | NA | NA | 1 | 1 | 0 | 1 | 1 | 1 | 21 |
| Madore et al. 1998 | 1 | 1 | 1 | 1 | 1 | 1 | 1 | NA | 1 | 1 | 1 | 1 | 1 | 1 | 1 | NA | NA | 0 | 1 | 1 | 0 | 1 | 0 | 1 | 1 | 1 | NA | NA | 1 | 1 | 1 | 1 | 1 | 0 | 21 |
| Strazzullo et al. 2001 | 1 | 1 | 1 | 1 | 0 | 0 | 0 | NA | 1 | 1 | 1 | 1 | 1 | 1 | 1 | NA | 1 | 0 | 1 | 0 | 0 | 1 | 0 | 1 | 1 | 1 | NA | NA | 1 | 1 | 1 | 1 | 1 | 1 | 19 |
| Gillen et al. 2005 | 0 | 1 | 1 | 1 | 1 | 1 | 1 | NA | 1 | 1 | 1 | 1 | 1 | 1 | 1 | NA | 0 | 0 | 1 | 1 | 0 | 1 | 0 | NA | 1 | 0 | NA | NA | 1 | 1 | 1 | 1 | 0 | 0 | 19 |
| Domingos et al. 2011 | 0 | 1 | 1 | 0 | 1 | 1 | 1 | NA | 0 | 1 | 1 | 1 | 1 | 1 | 1 | NA | 0 | 0 | 0 | 0 | 0 | 0 | 0 | NA | 1 | 1 | NA | NA | 1 | 1 | 1 | 1 | 1 | 0 | 17 |
| Ando et al. 2012(abstract only) | 1 | 1 | 0 | 1 | 1 | 1 | 0 | NA | 0 | 0 | 0 | 0 | 1 | 0 | 0 | NA | 0 | 0 | 0 | 0 | 0 | 0 | 0 | NA | 1 | 0 | NA | NA | 1 | 1 | 0 | 0 | 0 | 0 | 8 |
| Kittanamongkolchai et al. 2017 | 1 | 1 | 1 | 1 | 1 | 1 | 1 | 1 | 1 | 1 | 1 | 1 | 1 | 1 | 1 | NA | 1 | 0 | 1 | 1 | 1 | 1 | 0 | 1 | 1 | 1 | NA | NA | 1 | 1 | 1 | 1 | 1 | 1 | 22 |

Note: NA, not avaliable..
