# Supplementary material for: Nephrolithiasis and risk of hypertension: a meta-analysis of observational studies
Source: BMC Nephrol. 2017 Nov 29;18:344. doi: 10.1186/s12882-017-0762-8 (PMC5708110; doi:10.1186/s12882-017-0762-8)
Supplement: Supplementary file 2 — Sensitivity analysis. (DOC 28 kb) [file 12882_2017_762_MOESM2_ESM.doc]

Table S2 Sensitivity analysis

| Study omitted | OR | 95%CI | | I^2^ (%) | *P*^a^ |
| --- | --- | --- | --- | --- | --- |
| Madore et al. (1) 1998 (cohort) | 1.47 | 1.32 | 1.64 | 85.2 | <0.001 |
| Madore et al. (1) 1998 (cross-sectional) | 1.42 | 1.28 | 1.57 | 83.9 | <0.001 |
| Madore et al. (2) 1998 (cohort) | 1.45 | 1.31 | 1.62 | 85.6 | <0.001 |
| Madore et al. (2) 1998 (cross-sectional) | 1.46 | 1.29 | 1.65 | 80.6 | <0.001 |
| Strazzullo et al. 2001 | 1.41 | 1.29 | 1.55 | 84.6 | <0.001 |
| Gillen et al. 2005 | 1.43 | 1.30 | 1.57 | 85.5 | <0.001 |
| Domingos et al. 2011 | 1.34 | 1.27 | 1.40 | 37.7 | 0.129 |
| Ando et al. 2012 | 1.44 | 1.30 | 1.60 | 85.6 | <0.001 |
| Kittanamongkolchai et al. 2017 | 1.42 | 1.29 | 1.56 | 85.2 | <0.001 |

Abbreviations: OR, odds ratio; CI, confidence interval.

^a^ *P* value for heterogeneity among studies assessed with Cochran’s Q test.
